# Supplementary material for: Globally scalable glacier mapping by deep learning matches expert delineation accuracy
Source: Nat Commun. 2025 Jan 2;16:43. doi: 10.1038/s41467-024-54956-x (PMC11695715; doi:10.1038/s41467-024-54956-x)
Supplement: Supplementary file 1 — Supplementary Info [file 41467_2024_54956_MOESM1_ESM.pdf]

Supplementary information

Globally Scalable Glacier Mapping by Deep Learning Matches Expert Delineation Accuracy

Konstantin A. Maslov, Claudio Persello, Thomas Schellenberger, Alfred Stein

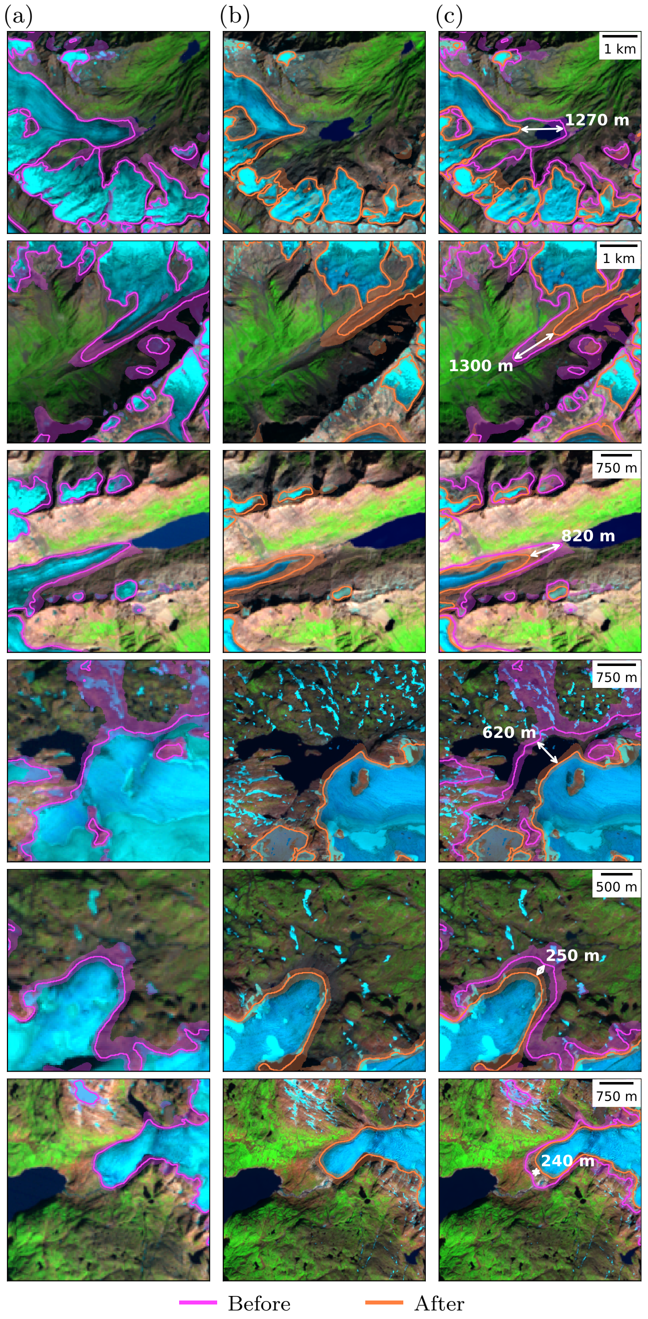

Supplementary Fig. 1 Closeups demonstrating decadal glacier changes mapped with GlaViTU: a before, **b** after and **c** both overlaid. Three upper rows: Aletsch complex (1998 vs 2023). Three lower rows: Hardangerjøkulen (1996 vs 2022). Polylines represent glacier outlines and semi-transparent polygons indicate 95% confidence bands. Landsat images courtesy of the U.S. Geological Survey. Copernicus Sentinel data 2022.

Supplementary Notes

Decadal glacier change mapping. Here we demonstrate the applicability of GlaViTU for the decadal mapping of glacier areal changes. We conducted two case studies at two sites—the Aletsch complex in the Swiss Alps and the Hardangerjøkulen in Southern Norway. For the Aletsch complex, we utilised Landsat 5 data from 1998 and SRTM (the same as in the independent acquisition test dataset), and Landsat 8 data from 2023 alongside Cop30DEM. For Hardangerjøkulen (not present in either the tile-based dataset or the independent acquisition test dataset), we used Landsat 5 data from 1996 and SRTM, followed by Sentinel-2 imagery from 2022 and Cop30DEM. In both cases, the glaciers were mapped with the GlaViTU model trained with region encoding. During inference, bias optimisation was used as well.

The Aletsch complex experienced a notable decrease in area over the 25 years. In 1998, the glaciers covered an area of 340.01 km^2, with uncertainty ranging from 253.12 to 439.83 km^2 as indicated by the 95% confidence bands derived from the model predictions. By 2023, the area had diminished to 257.48 km^2 (179.53–350.43 km^2). Similarly, Hardangerjøkulen showed a reduction in area from 77.01 km^2 (69.65–90.09 km^2) to 64.48 km^2 (58.05–71.88 km^2). Supplementary Figure 1 shows some closeups of the glacier outlines and the confidence bands for the initial and final years of the study periods.

The case studies presented here provide concrete evidence of GlaViTU utility in tracking and analysing glacier area changes over decades as well as an additional qualitative assessment of the robustness and accuracy of our method. These results enable the future scaling of GlaViTU for global glacier monitoring.

Comparison with SGI2016. As an additional validation step, we compared the outputs of GlaViTU with an inventory derived manually from sub-meter resolution aerial imagery, namely, Swiss Glacier Inventory 2016 (SGI2016)[1]. We took an area from the Swiss Alps (spanning 7°41′30″ N–9°07′14″ N, 45°57′39″ E–46°56′48″ E) and collected corresponding satellite data from 2016. These data included one Sentinel-2 scene and four Sentinel-1 scenes forming two interferometric pairs in both ascending and descending orbital paths. We also used Cop30DEM tiles covering the same area of interest. From the Sentinel-1 pairs, we extracted backscatter and InSAR coherence to match the Optical+DEM+InSAR data track.

We fed these data into the GlaViTU model trained

Supplementary Table 1 Glacier delineation accuracy for a subsample of 15 glaciers from SGI2016[1]. The percentage of debris cover is reported as the fraction of glacier area not classified by the band ratio method within the reference outlines. Ice divides were copied from the reference data.

Glacier	Debris coverage, %	Pixel size, m	Area, km²		Area deviation,		Distance deviation, m			IoU
			Reference	Predicted	km²	%	Mean	Median	95th percentile	
Lötschegletscher	39.70	10	0.731	0.691	–0.040	–5.54	20.02	12.49	70.46	0.852
Gamchigletscher	65.16	10	1.065	0.953	–0.112	–10.54	45.64	25.77	158.52	0.626
Wallenburfirn	33.53	10	1.387	0.965	–0.422	–30.44	62.04	14.68	289.95	0.661
Chelengletscher	8.04	10	1.764	1.701	–0.063	–3.54	12.41	5.15	58.45	0.929
Weissmiesgletscher	45.45	10	1.920	1.705	–0.215	–11.19	39.06	12.49	178.50	0.813
Alpjergletscher	3.80	10	2.091	2.112	+0.022	+1.04	15.47	6.63	60.54	0.917
Breithorngletscher	32.14	10	2.529	2.569	+0.040	+1.59	14.00	7.89	49.91	0.922
Oberaletschgletscher	27.59	10	3.949	3.591	–0.358	–9.06	33.05	11.63	137.87	0.826
Langgletscher	14.83	10	8.000	7.643	–0.357	–4.46	31.34	8.61	135.92	0.902
Kanderfirn N	8.50	10	11.961	12.027	+0.066	+0.55	20.65	8.52	79.62	0.960
Hüfifirn	5.94	10	12.622	12.112	–0.510	–4.04	19.47	8.12	80.83	0.948
Triftgletscher	4.30	10	14.548	14.378	–0.171	–1.18	18.31	6.37	83.79	0.954
Rhonegletscher	5.60	10	14.626	14.038	–0.588	–4.02	25.58	8.22	125.52	0.949
Unteraargletscher	41.66	10	22.681	21.311	–1.371	–6.04	28.26	10.01	123.66	0.897
Aletschgletscher	11.31	10	78.436	77.014	–1.421	–1.81	20.82	7.62	94.11	0.952

globally on the Optical+DEM+InSAR data track without any adjustments to the model. We achieved a nice correspondence between the predicted outlines and the reference data (IoU = 0.865) comparable to those presented previously. The total area was underestimated by −6.39%, varying from −29.34% to 12.84% if taking into account 95%-confidence bands. In addition, 74.74% of misclassified pixels were within the confidence bands, with $ECE_{100} = 0.00231$, indicating strong calibration performance. For a more detailed analysis, we selected a subsample of 15 glaciers and presented a comparison in terms of area and distance deviations in Supplementary Table 1 (similar to Supplementary Table 6). The area and distance deviations were also consistent with previously presented results, with the exception of one glacier—Wallenburfirn where the area error was considerably higher (−30.44%) due to missclassified debris-covered tongue. Part of the tongue was within the confidence bands, and the relative area deviation would be −1.51% if the bands were included. Although not directly comparable in this case, this discrepancy is still in the range of human expert uncertainty when interpreting satellite images as reported by Paul et al.[2].

It is important to note that SGI2016 is derived from very high-resolution data, which is of inherently higher quality than the satellite data used in our study, making a direct comparison challenging and potentially less fair. Additionally, SGI2016 outlines are also subject to uncertainty as reported in a round-robin experiment with five experts[1]. The distance and area deviations can be up to 100 meters and 23.8%, respectively, while one standard deviation of area for a glacier lies between 0.3% and 7.8%. Notably, SGI2016 may be influenced by subjective choices made by annotators, particularly regarding the inclusion of lateral moraines, which explains why area deviations from GlaViTU tend to be negative in our results. Overall, this analysis incorporates an additional validation using a completely independent dataset, which provides further insights into the effectiveness and robustness of GlaViTU.

Comparison with baselines. In the previous study[3], we compared GlaViTU with SETR-B/16[4], ResU-Net[5,6] and TransU-Net[7], where it outperformed these three baseline models. Here, we compared GlaViTU with DeepLabv3+[8] with the ResNeSt-101 backbone[9], often used as a state-of-the-art baseline in computer vision studies[10]. We refer to this model as DeepLabv3+/ResNeSt-101. The fusion block (Supplementary Figure 8) was attached to DeepLabv3+/ResNeSt-101 as well instead of the original ResNeSt stem to ensure a fair comparison of the models.

We compared GlaViTU with DeepLabv3+/ResNeSt-101 for all regions (Supplementary Table 2). GlaViTU achieved an average IoU of 0.894, 1.7% higher than the IoU of 0.877 of DeepLabv3+/ResNeSt-101, consistently outperforming the latter across all regions. Both models performed very well for regions with predominantly snow and ice conditions such as the Southern Andes (IoU = 0.952 of GlaViTU vs. IoU = 0.948 of DeepLabv3+/ResNeSt-101), Antarctica (0.949 vs. 0.944), Greenland (0.937 vs. 0.930) and Svalbard (0.936 vs. 0.931). A drop in performance is seen for both models for areas with significant debris cover, with the worst performance in High-Mountain Asia (0.774 vs. 0.747), but also for Caucasus (0.862 vs. 0.853) and the Alps (0.844 vs. 0.835). The largest differences in performance occurred for Scandinavia (0.908 vs. 0.827), low latitudes (0.903 vs. 0.872) and High-Mountain Asia (0.774 vs. 0.747).

Several comparisons are shown in Supplementary Figure 2. A major advantage of GlaViTU was its better, although not perfect, performance in identifying debris-covered ice (Supplementary Figure 2 a,b,c,d). Moreover, GlaViTU produced fewer false positives compared to DeepLabv3+/ResNeSt-101 for specific cases. For instance, it yielded fewer incorrect results for water bodies (Supplementary Figure 2 e). Similarly, GlaViTU also had fewer false positives for bare rocks that DeepLabv3+/ResNeSt-101 tended to confuse with debris (Supplementary Figure 2 f). In specific conditions, GlaViTU was also better at handling ice mélange (Supplementary Figure 2 g). To sum up, the GlaViTU model consistently outperforms

Supplementary Table 2 Comparison of the glacier mapping models on the tile-based test dataset and Optical+DEM data. Region abbreviations: ALP (the Alps), ANT (Antarctica), AWA (Alaska and Western America), CAU (Caucasus), GRL (Greenland), HMA (High-Mountain Asia), TRP (low latitudes), NZL (New Zealand), SAN (the Southern Andes), SCA (Scandinavia), SVAL (Svalbard).

Model	IoU[a] of different regions											Average IoU
	ALP	ANT	AWA	CAU	GRL	HMA	TRP	NZL	SAN	SCA	SVAL	
DeepLabv3+/ResNeSt-101	0.835	0.944	0.909	0.853	0.930	0.747	0.872	0.850	0.948	0.827	0.931	0.877
GlaViTU	**0.844**	**0.949**	**0.912**	**0.862**	**0.937**	**0.774**	**0.903**	**0.860**	**0.952**	**0.908**	**0.936**	**0.894**

[a] The best IoU values are in bold.

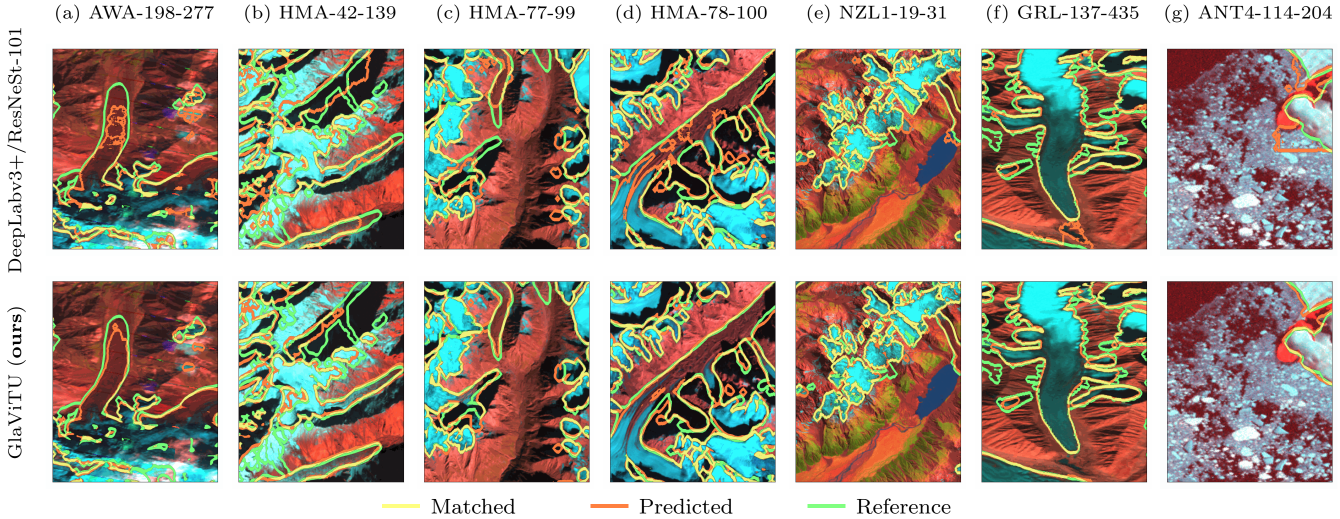

Supplementary Fig. 2 Comparison of the results derived with DeepLabv3+/ResNeSt-101 and GlaViTU on the tile-based test dataset and Optical+DEM data: a–d debris-covered ice, **e** a water body, **f** surrounding rocks and **g** ice mélange. The satellite images are presented in a false colour composition (R: SWIR$_{\approx 2.2\mu\text{m}}$, G: NIR, B: R). Landsat images courtesy of the U.S. Geological Survey. Copernicus Sentinel data 2019.

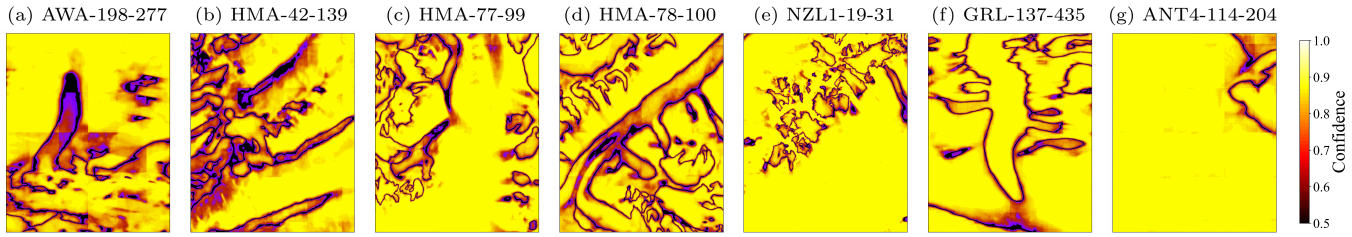

Supplementary Fig. 3 Calibrated confidence estimates for the classification examples presented in the bottom panel of Figure 2.

DeepLabv3+/ResNeSt-101 as well across all regions, producing good-quality results for the majority of the images.

Confidence-based IoU estimation. In the absence of reference data, assessing the quality of glacier mapping poses a significant challenge. Traditional metrics like precision, recall and IoU rely on groundtruth data for evaluation. Obtaining such reference data can be impractical or costly in real-world scenarios. While Equation (4) establishes a connection between the predicted confidence and accuracy, it is not very informative as an aggregated metric in the context of glacier mapping because of the class imbalance. To address this issue, we propose a simple relation that leverages the model predictions and calibrated confidence scores to estimate IoU. Please note that this relation is not employed in other sections of the paper, where we report IoU metrics based on available reference data. It serves as an auxiliary tool to assess mapping quality in the absence of reference data, complementing rather than replacing the traditional IoU metric, and is useful for getting preliminary estimates of the quality of new glacier inventories generated with our method.

Given that for a particular class:

$$\text{IoU} = \frac{\text{TP}}{n_\text{p} + \text{FN}}, \quad \text{Accuracy} = \frac{\text{TP} + \text{TN}}{n_\text{p} + n_\text{n}}, \tag{1}$$

IoU can be defined as:

$$\text{IoU} = 1 - (1 - \text{Accuracy}) \cdot \frac{n_\text{p} + n_\text{n}}{n_\text{p} + \text{FN}}, \tag{2}$$

where TP are true positives, FN are false negatives, TN are true negatives, and n_p and n_n are the total numbers of positives and negatives, respectively. The aim is to find good approximations for Accuracy and FN, the only unknown terms without the reference data.

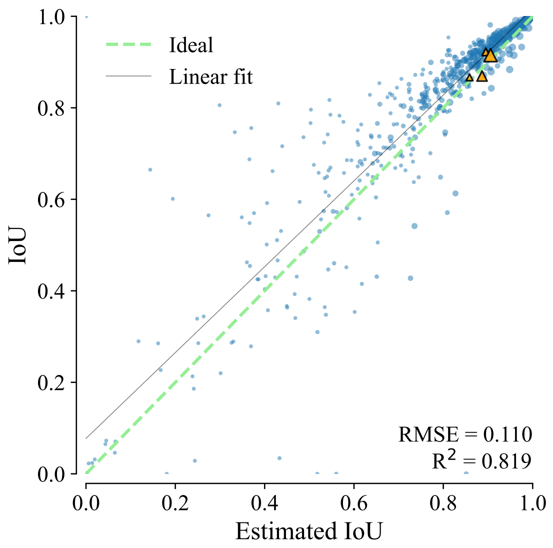

Supplementary Fig. 4 IoU values estimated with Supplementary Equation (5) versus the observed values for the test tiles (556 blue dots) and the independent acquisition test data (four orange triangles). Marker size is proportional to n_p. Source data are provided as a Source Data file.

We then introduce two approximations:

$$\text{FN} \approx (1 - \text{Accuracy}) \cdot n_\text{n} \,, \tag{3}$$

$$\text{Accuracy} \approx \max\left(\bar{\bar{\gamma}}, \frac{1}{C}\right) \,, \tag{4}$$

where $\bar{\bar{\gamma}}$ is the mean predicted confidence, and C is the number of classes. Supplementary Equation (3) is based on a common-sense relationship between FN, Accuracy and n_n: FN $= n_\text{n}$ if Accuracy $= 0$ and FN $= 0$ if Accuracy $= 1$, assuming that this relationship is linear. Supplementary Equation (4) establishes a relationship between Accuracy and $\bar{\bar{\gamma}}$ due to confidence calibration and sets the lower bound for Accuracy under the assumptions that the mapping model is better than random guessing and the classes are balanced for low confidence values.

Finally, our relation was defined as follows:

$$\text{IoU} \approx 1 - \left(1 - \max\left(\bar{\bar{\gamma}}, \tfrac{1}{C}\right)\right) \cdot \frac{n_\text{p} + n_\text{n}}{n_\text{p} + \left(1 - \max\left(\bar{\bar{\gamma}}, \frac{1}{C}\right)\right) \cdot n_\text{n}} \,. \tag{5}$$

We compared the correspondence between the estimated IoU values from Supplementary Equation (5) and actual IoU values for two datasets—the testing subset tiles and the independent acquisition test data. Supplementary Figure 4 depicts the corresponding scatter plot. Our findings showed a strong agreement between the estimated and actual IoU values. The root mean square error between the estimated and actual IoU values was as low as 0.110, indicating a high degree of accuracy in our predictions. Furthermore, the coefficient of determination between the two sets of IoU values was 0.819, suggesting that our confidence-based estimates capture a substantial portion of the variability in the actual IoU values. We observed a high correlation between actual IoU and estimated IoU values, as evidenced by the Pearson correlation coefficients ranging between 0.873 and 0.948 (reported as the 2.5$^\text{th}$ and 97.5$^\text{th}$ percentiles obtained from bootstrapping). Nevertheless, our confidence-based IoU estimation relation tended to slightly underestimate the actual IoU values by an average margin of 3%. While this discrepancy may be viewed as a limitation, we consider it to be within an acceptable range.

There remains room for improvement. One potential avenue for refinement is the parameterisation of the proposed relation. For example, one could consider replacing Supplementary Equation (3) with

$$\text{FN} \approx (1 - \text{Accuracy})^\beta \cdot n_\text{n} \tag{6}$$

and fitting the parameter $\beta \geq 0$ with the observed values, thus making the relation more flexible to potential nonlinearities. Alternatively, using data-driven methods such as linear regression could allow for adjustments that result in more accurate IoU estimates. For brevity, we omitted these options here. Besides, they might compromise the simplicity and elegance of the original parameter-free relation that does not require fitting.

While our approach has demonstrated success within the context of glacier mapping, its generalisability to other datasets and problem domains remains an open question. Assumptions embedded in Supplementary Equations (3) and (4) may not hold in different scenarios or for multi-class problems, necessitating further investigation. Overall, Supplementary Equation (5) offers a practical and parameter-free solution to estimate IoU and assess glacier mapping quality in our experimental setup when reference data is lacking.

Ice divides. In this study, we employed the algorithm proposed by Kienholz et al.[11] to reconstruct ice divides. We leverage the tools of WhiteboxTools[12] and Pysheds[13] for an open-source implementation to increase transparency, reproducibility and accessibility for the scientific community.

The quality of the reconstructed ice divides was estimated with two distance-based metrics similar to PoLiS[14]. The first metric calculates the average distance from points in the reconstructed ice divides to the reference, while the second metric calculates the distance from points in the reference to the reconstructed ice divides. The metrics are defined as follows:

$$\overline{\rho(p_\text{rc}, \text{rf})} = \frac{1}{|\{p \in \text{rc}\}|} \cdot \sum_{\{p \in \text{rc}\}} \rho\,(p, \text{rf}) \,, \tag{7}$$

$$\overline{\rho(p_\text{rf}, \text{rc})} = \frac{1}{|\{p \in \text{rf}\}|} \cdot \sum_{\{p \in \text{rf}\}} \rho\,(p, \text{rc}) \,, \tag{8}$$

where p is a point of a polyline, rf and rc are the reference and reconstructed ice divides, respectively, and ρ stands for the Euclidean distance. Points p were sampled every 10 metres from both rc and rf for the calculations. We used the independent acquisition test dataset to evaluate the algorithm, the only difference is that we extracted the Jostedalbreen glacier from Southern Norway to assess the performance specifically on ice caps. We calibrated the algorithm for every region with Bayesian optimization by

Supplementary Table 3 Ice divides quality assessment.

Area	Terrain	DEM	$\overline{\rho(p_{\mathrm{rc}}, \mathrm{rf})}$, m	$\overline{\rho(p_{\mathrm{rf}}, \mathrm{rc})}$, m	$\frac{1}{2} \cdot \left[\overline{\rho(p_{\mathrm{rc}}, \mathrm{rf})} + \overline{\rho(p_{\mathrm{rf}}, \mathrm{rc})}\right]$, m
Swiss Alps	Mountainous	SRTM	109	389	249
Jostedalsbreen	Ice cap	Cop30DEM	142	117	130
Alaska	Mountainous	AW3D30	453	271	362
Southern Canada	Mountainous	AW3D30	891	268	580

minimizing the average of Supplementary Equations (7) and (8).

Supplementary Table 3 summarises the results of the implemented algorithm for ice divides delineation after calibrating its parameters. Overall, the average spatial deviation between the reference data and the reconstructed ice divides was roughly in the range of hundreds of meters, falling between 100 and 600 m. Remarkably, the lowest discrepancy was observed for Jostedalsbreen, an ice cap, while one could expect that reconstructing ice divides for mountainous regions should be less challenging due to the prominent elevation features and high slopes. Also, the distances from the reconstructed ice divides to the reference exhibited higher errors in Alaska and Southern Canada, which can indicate the tendency of the algorithm to split ice complexes in these areas into smaller glaciers.

The implemented ice divides delineation routine, while not perfect, represents an important step in the automation of glacier inventory generation, allowing building a workflow that starts from remotely sensed data and finishes in individual glacier outlines. This automated approach has the potential to reduce the manual effort required for glacier mapping and enables us to scale up the mapping efforts considerably. However, the question of how best to reconstruct ice divides remains open. One potential avenue for improvement is the incorporation of velocity data derived from InSAR, as done by, e.g., Strozzi et al.[15], or from feature tracking, as in, e.g., Thakur et al.[16]. Such velocity data can provide valuable insights into the flow dynamics of glaciers and help refine the delineation of ice divides in regions where this information is available. Another consideration is the possibility of directly copying ice divides from RGI[17] as proposed by Racoviteanu et al.[18], which would ensure consistency in individual glacier outlines from year to year, a crucial factor for conducting surface mass balance time series analysis. This approach also has limitations, particularly in cases where RGI-derived ice divides may not accurately reflect the actual dynamics of the glaciers. The reference data used for ice divides delineation are subject to uncertainties. Many of these reference data are likely derived from DEMs and may not necessarily represent the true ice divides. Thus, there is a need for continued research in the direction of ice divides delineation.

Supplementary Figures and Tables

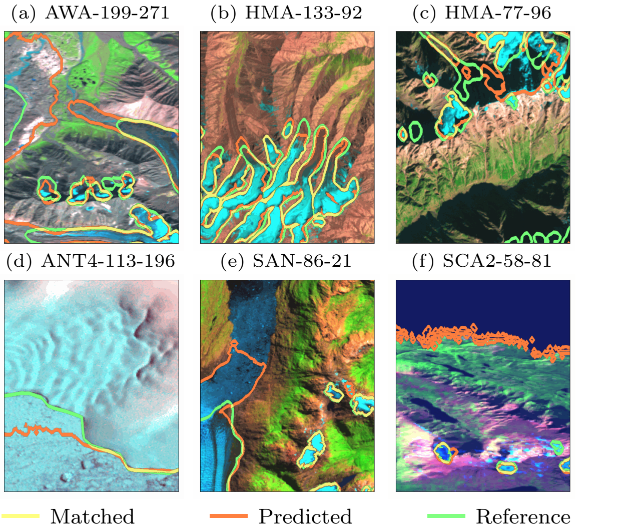

Supplementary Fig. 5 Failed semantic segmentation examples with GlaViTU from the Optical+DEM data track on the tile-based test dataset: a, b debris (we suspect errors in the reference data on the left side of **b**, and the actual glacier terminus should be located farther to the north), **c** shadows, **d, e** ice mélange and **f** artefacts at coastlines. The satellite images are presented in a false colour composition (R: $\text{SWIR}_{\approx 2.2\mu m}$, G: NIR, B: R). Landsat images courtesy of the U.S. Geological Survey.

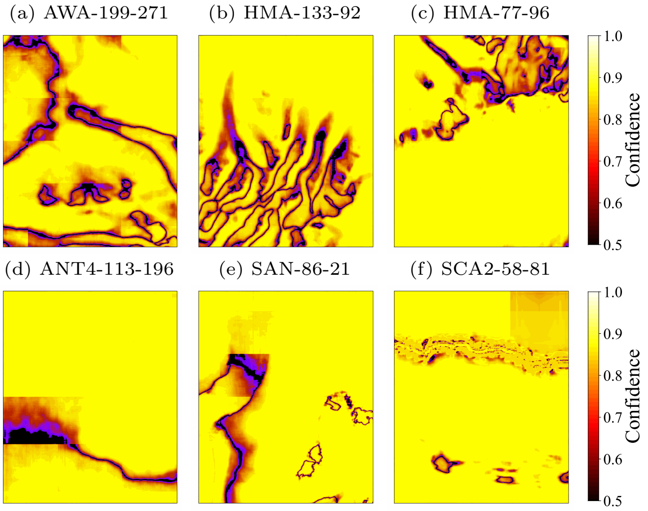

Supplementary Fig. 6 Calibrated confidence estimates for the examples of failed semantic segmentation presented in Figure 5.

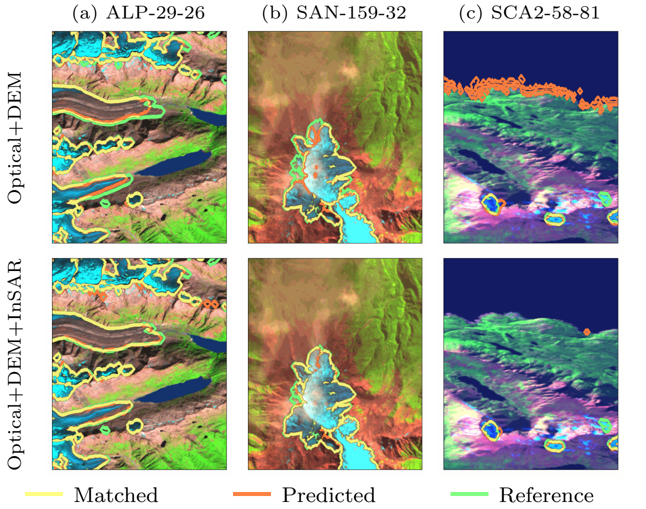

Supplementary Fig. 7 Comparison of the results derived with the Optical+DEM and Optical+DEM+InSAR data tracks on the tile-based test dataset: a glacier termini, **b** cloud occlusion and **c** artefacts at coastlines. The satellite images are presented in a false colour composition (R: $\text{SWIR}_{\approx 2.2\mu m}$, G: NIR, B: R). Copernicus Sentinel data 2015. Landsat images courtesy of the U.S. Geological Survey.

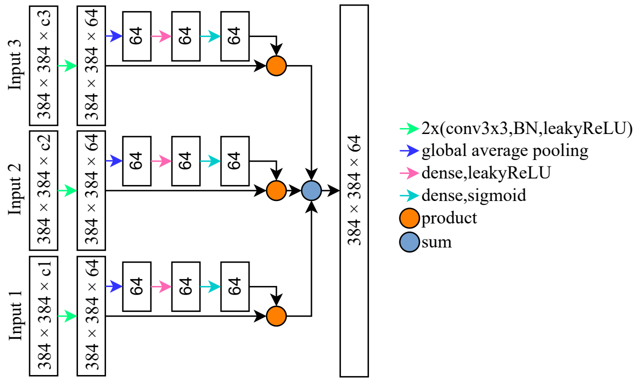

Supplementary Fig. 8 New design of the fusion block. The case of three inputs.

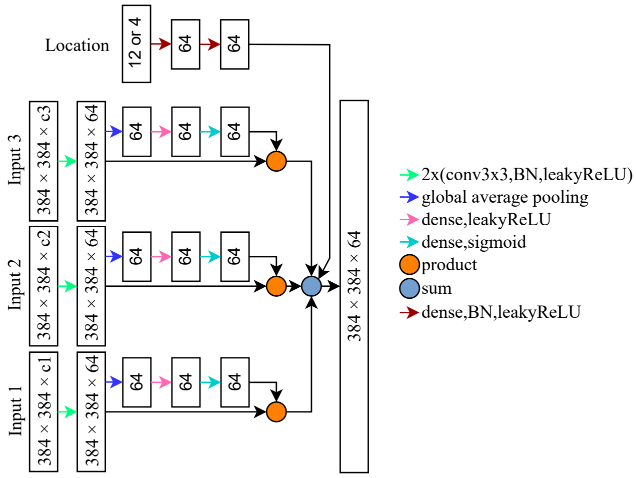

Supplementary Fig. 9 Fusion block with location encoding. The case of three inputs. The size of the location vector can be 12 or 4 for region or coordinate encoding, respectively.

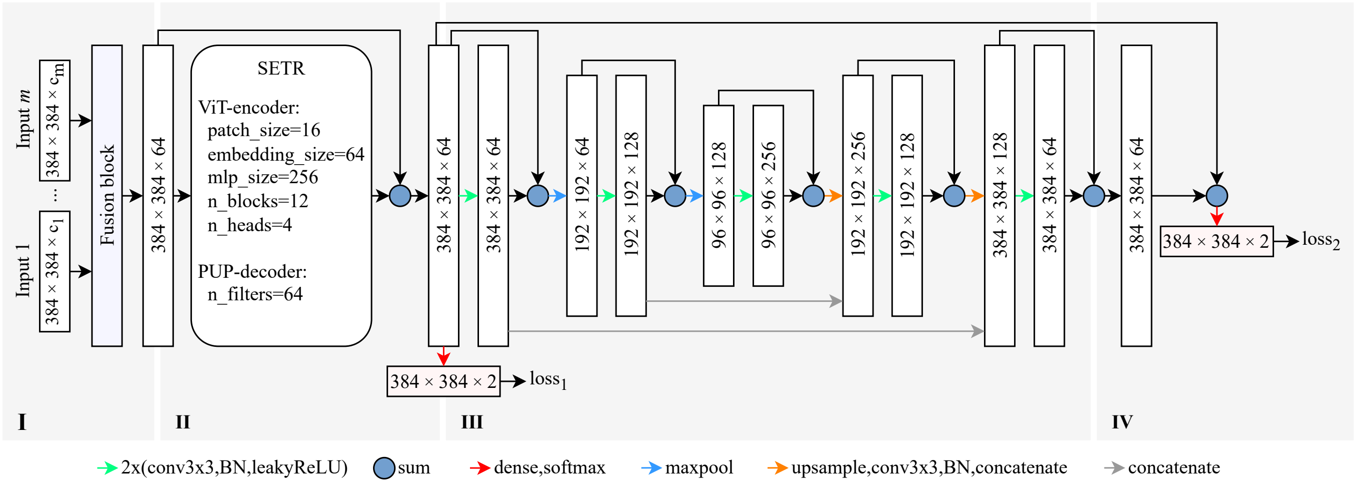

Supplementary Fig. 10 Glacier-VisionTransformer-U-Net (GlaViTU). Boxes and numbers in them represent tensors and their shapes in the height × width × channels format, and arrows indicate operations and data flow. GlaViTU **I** fuses multi-modal inputs such as satellite images and elevation models, **II** extracts global features with a vision transformer, **III** refines local features with a convolutional subnet and **IV** yields the final classification map.

Supplementary Table 4 Comparison of the strategies towards global glacier mapping on the tile-based test dataset. Region abbreviations: ALP (the Alps), ANT (Antarctica), AWA (Alaska and Western America), CAU (Caucasus), GRL (Greenland), HMA (High-Mountain Asia), TRP (low latitudes), NZL (New Zealand), SAN (the Southern Andes), SCA (Scandinavia), SVAL (Svalbard).

Strategy	IoU[a] of different regions											Average IoU
	ALP	ANT	AWA	CAU	GRL	HMA	TRP	NZL	SAN	SCA	SVAL	
Global	0.844	0.949	**0.912**	0.862	0.937	0.774	0.903	0.860	0.952	0.908	0.936	0.894
Regional	**0.876**	0.950	0.898	0.855	**0.943**	**0.789**	0.905	0.861	**0.965**	**0.939**	0.937	**0.902**
Finetuning	0.866	0.952	0.873	**0.873**	0.940	0.787	**0.908**	**0.874**	0.960	0.937	**0.939**	0.901
Region encoding	0.865	**0.952**	0.911	0.867	0.940	0.775	0.908	0.867	0.956	0.891	0.936	0.897
Coordinate encoding	0.857	0.950	0.906	0.862	0.935	0.763	0.907	0.865	0.953	0.892	0.936	0.893

[a] The best IoU values are in bold.

Supplementary Table 5 Glacier detection accuracy on independent acquisition test dataset. A reference polygon and a predicted polygon were considered matched (true positive) if their intersection area consisted more that 50% of both of them individually. Ice divides were copied from the reference data.

Region	Metric	Glacier size, km^2				Total
		≤ 0.1	0.1–1	1–10	> 10	
GlaViTU						
Swiss Alps	Precision	0.403	0.795	0.943	1.00	0.699
	Recall	0.337	0.881	0.971	1.00	0.693
	F1	0.367	0.836	0.957	1.00	0.696
Southern Norway (SCA1)	Precision	0.388	0.912	0.993	1.00	0.639
	Recall	0.249	0.951	0.993	1.00	0.495
	F1	0.303	0.931	0.993	1.00	0.558
Alaska	Precision	0.242	0.726	0.899	1.00	0.592
	Recall	0.183	0.810	0.929	1.00	0.554
	F1	0.208	0.766	0.914	1.00	0.572
Southern Canada	Precision	0.042	0.616	0.978	1.00	0.427
	Recall	0.438	0.881	0.993	1.00	0.883
	F1	0.077	0.725	0.986	1.00	0.575

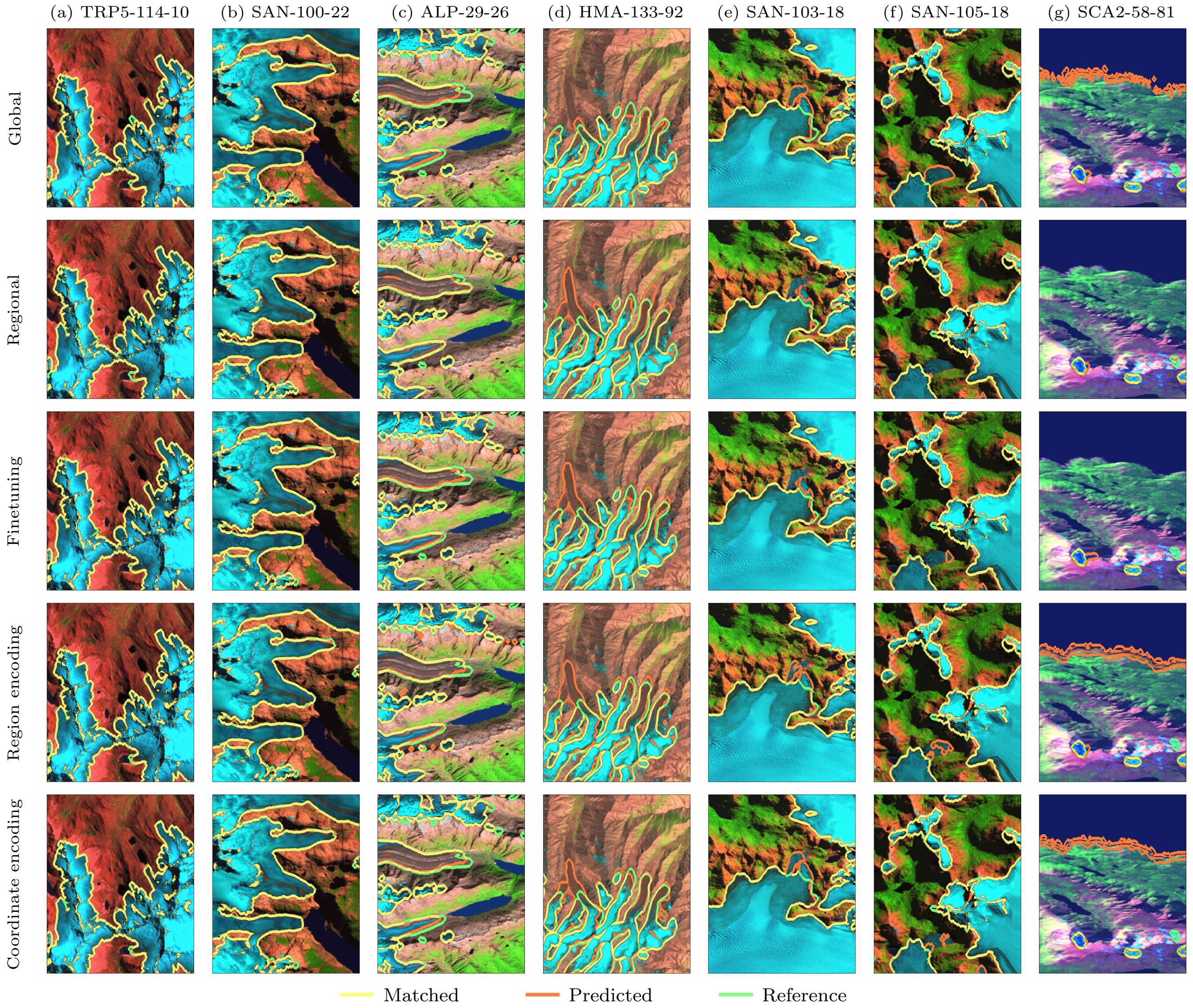

Supplementary Fig. 11 Comparison of the results derived with different strategies on the tile-based test dataset and Optical+DEM data: a, b mostly clean ice, **c, d** debris-covered ice, **e, f** ice mélange and **g** artefacts on coastlines. The satellite images are presented in a false colour composition (R: $SWIR_{\approx 2.2\mu m}$, G: NIR, B: R). Landsat images courtesy of the U.S. Geological Survey. Copernicus Sentinel data 2015.

Supplementary Table 6 Glacier delineation accuracy for a subsample of 17 representative glaciers from the independent acquisition test dataset. The percentage of debris cover is reported as the fraction of glacier area not classified by the band ratio method within the reference outlines. Ice divides were copied from the reference data.

Glacier	Debris coverage, %	Pixel size, m	Area, km^2 Reference	Predicted	Area deviation, km^2	%	Distance deviation, m Mean	Median	95th percentile	IoU
Brattbreen	11.58	10	0.153	0.135	–0.018	–11.90	37.25	5.00	226.75	0.865
Gamchigletscher	76.09	30	1.458	1.549	+0.090	+6.19	44.69	32.02	123.96	0.797
Tundraskarsbreen	3.20	10	3.231	3.163	–0.068	–2.12	7.95	5.00	32.02	0.969
Langgletscher	18.94	30	9.115	8.973	–0.142	–1.55	52.83	30.00	183.78	0.883
Dorothy glacier	9.33	30	9.185	9.995	+0.809	+8.81	40.92	26.12	142.33	0.870
Oberaletschgletscher	35.92	30	18.938	19.644	+0.706	+3.73	44.22	30.00	152.13	0.858
Kilippi glacier	3.14	30	22.397	22.687	+0.291	+1.30	13.87	10.01	45.63	0.967
Unteraargletscher	36.40	30	24.632	24.831	+0.199	+0.81	53.79	32.15	205.31	0.869
Tonsina glacier	13.92	30	40.688	39.657	–1.031	–2.53	50.62	22.87	251.40	0.932
Scimitar glacier	16.73	30	41.038	47.692	+6.654	+16.21	52.30	26.89	188.85	0.854
Tunsbergdalsbreen	2.02	10	46.045	45.800	–0.245	–0.53	18.91	5.00	109.66	0.984
Stephens glacier	25.53	30	49.793	46.794	–3.000	–6.02	46.26	24.28	174.88	0.915
Tiedemann glacier	16.90	30	58.890	63.823	+4.933	+8.38	47.38	28.55	160.81	0.891
Aletschgletscher	9.55	30	82.278	83.934	+1.656	+2.01	39.54	30.31	103.34	0.937
Marcus Baker glacier	23.51	30	173.756	166.776	–6.979	–4.02	45.03	20.01	200.08	0.925
Matanuska glacier	17.54	30	319.142	299.263	–19.878	–6.23	59.93	15.43	209.33	0.927
Klinaklini glacier	1.84	30	469.910	474.177	+4.267	+0.91	41.33	11.51	118.16	0.970

Supplementary Table 7 Comparison of the feature sets on the tile-based test dataset. The metrics are reported for global models. Note that the metrics are calculated for the subregions where data availability is not consistent within one region. Region abbreviations: ALP (the Alps), ANT (Antarctica), AWA (Alaska and Western America), CAU (Caucasus), GRL (Greenland), HMA (High-Mountain Asia), TRP (low latitudes), NZL (New Zealand), SAN (the Southern Andes), SCA (Scandinavia), SVAL (Svalbard).

Feature set	IoU[a] of different subregions													
	ALP	ANT	AWA	CAU	GRL	HMA	TRP1	TRP2	NZL	SAN1	SAN2	SCA1	SCA2	SVAL
Optical+DEM	0.844	0.949	0.912	**0.862**	0.937	**0.774**	0.817	**0.903**	0.860	0.874	**0.958**	0.945	0.836	0.936
Optical+DEM+thermal	—	**0.951**	**0.915**	0.846	**0.937**	0.756	0.805	0.895	—	0.863	0.956	**0.946**	0.857	—
Optical+DEM+InSAR	**0.873**	—	—	—	—	—	**0.872**	—	**0.862**	**0.890**	—	—	**0.909**	**0.939**

[a] The best IoU values are in bold.

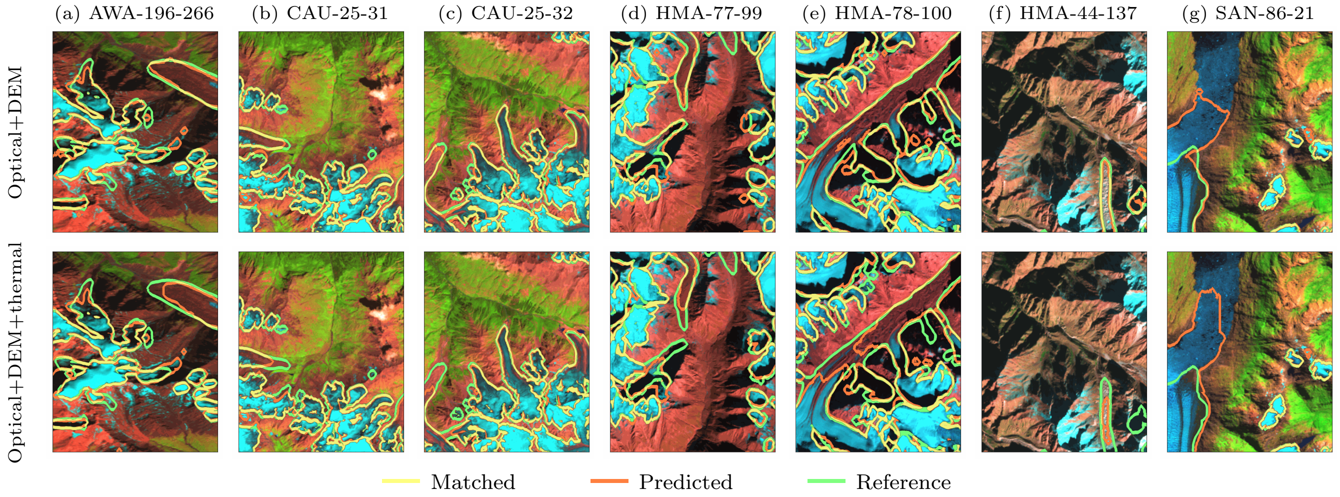

Supplementary Fig. 12 Comparison of the results derived with the Optical+DEM and Optical+DEM+thermal data tracks on the tile-based test dataset: a–f debris and **g** ice mélange. The satellite images are presented in a false colour composition (R: $SWIR_{\approx 2.2\mu m}$, G: NIR, B: R). Landsat images courtesy of the U.S. Geological Survey.

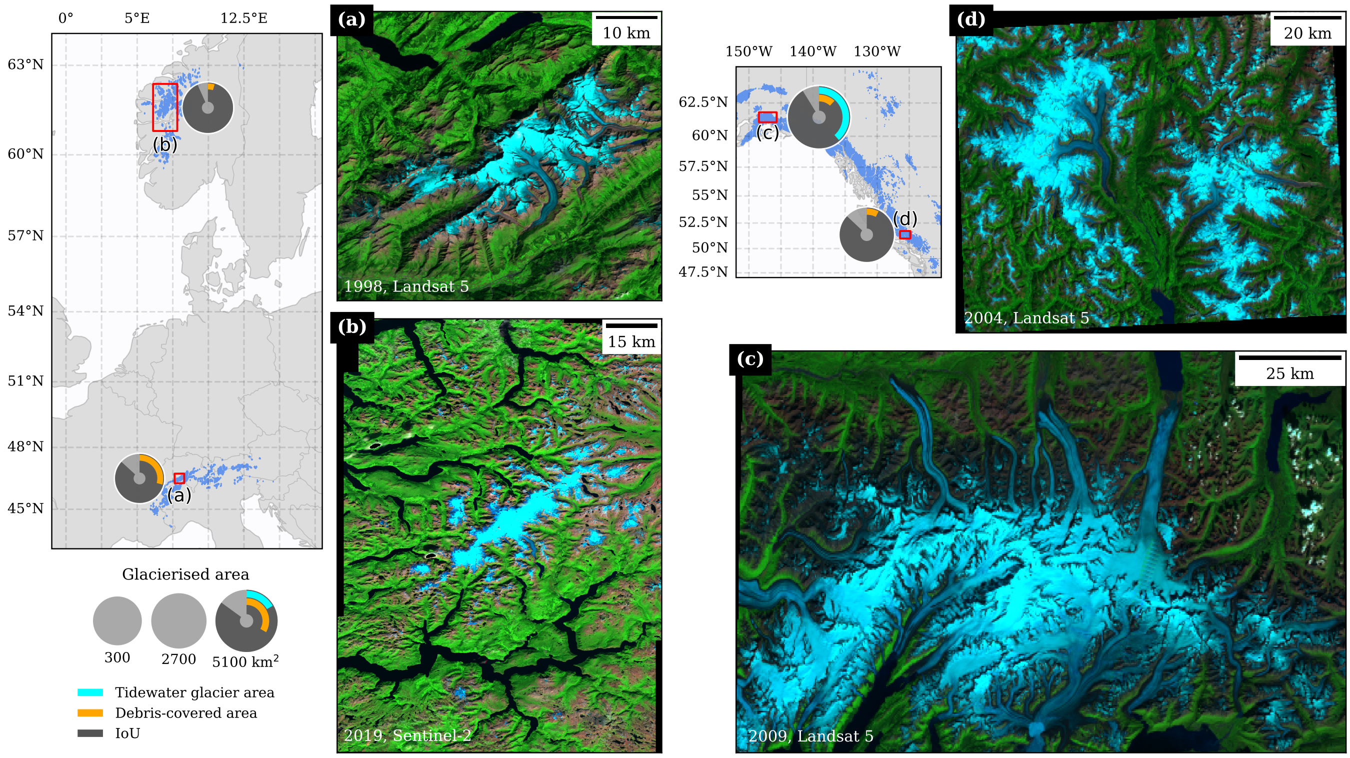

Supplementary Fig. 13 Independent acquisition test dataset: a the Swiss Alps, **b** Southern Norway, **c** Alaska and **d** Southern Canada. The satellite images are presented in a false colour composition (R: $SWIR_{\approx 2.2\mu m}$, G: NIR, B: R). The glacier outlines and tidewater glacier areas are based on RGI7.0[17]. Debris coverage is adapted from Herreid and Pellicciotti[19]. The IoU values are presented for the GlaViTU model trained with region encoding on Optical+DEM data after bias optimisation. Landsat images courtesy of the U.S. Geological Survey. Copernicus Sentinel data 2019. Source data are provided as a Source Data file.

References

1. Linsbauer, A. *et al.* The New Swiss Glacier Inventory SGI2016: From a Topographical to a Glaciological Dataset. *Frontiers in Earth Science* **9,** 704189. ISSN: 22966463 (Oct. 2021).
2. Paul, F. *et al.* On the accuracy of glacier outlines derived from remote-sensing data. *Annals of Glaciology* **54,** 171–182. ISSN: 02603055 (July 2013).
3. Maslov, K. A., Persello, C., Schellenberger, T. & Stein, A. *GLAVITU: A Hybrid CNN-Transformer for Multi-Regional Glacier Mapping from Multi-Source Data* in *IGARSS 2023 - 2023 IEEE International Geoscience and Remote Sensing Symposium* (2023), 1233–1236.
4. Zheng, S. *et al. Rethinking Semantic Segmentation from a Sequence-to-Sequence Perspective with Transformers* in *Proceedings of the IEEE Computer Society Conference on Computer Vision and Pattern Recognition* (IEEE Computer Society, Dec. 2020), 6877–6886. ISBN: 9781665445092. https://arxiv.org/abs/2012.15840v3.
5. Ronneberger, O., Fischer, P. & Brox, T. U-Net: Convolutional Networks for Biomedical Image Segmentation. *Lecture Notes in Computer Science (including subseries Lecture Notes in Artificial Intelligence and Lecture Notes in Bioinformatics)* **9351,** 234–241. ISSN: 16113349. https://arxiv.org/abs/1505.04597v1 (May 2015).
6. He, K., Zhang, X., Ren, S. & Sun, J. *Deep Residual Learning for Image Recognition* in *Proceedings of the IEEE Computer Society Conference on Computer Vision and Pattern Recognition* **2016-December** (IEEE Computer Society, Dec. 2015), 770–778. ISBN: 9781467388504. https://arxiv.org/abs/1512.03385v1.
7. Chen, J. *et al.* TransUNet: Transformers Make Strong Encoders for Medical Image Segmentation. https://arxiv.org/abs/2102.04306v1 (Feb. 2021).
8. Chen, L. C., Zhu, Y., Papandreou, G., Schroff, F. & Adam, H. Encoder-Decoder with Atrous Separable Convolution for Semantic Image Segmentation. *Lecture Notes in Computer Science* **11211 LNCS,** 833–851. ISSN: 16113349. https://arxiv.org/abs/1802.02611v3 (Feb. 2018).
9. Zhang, H. *et al.* ResNeSt: Split-Attention Networks. *IEEE Computer Society Conference on Computer Vision and Pattern Recognition Workshops* **2022-June,** 2735–2745. ISSN: 21607516. https://arxiv.org/abs/2004.08955v2 (Apr. 2020).
10. Strudel, R., Garcia, R., Laptev, I. & Schmid, C. *Segmenter: Transformer for Semantic Segmentation* in *Proceedings of the IEEE International Conference on Computer Vision* (Institute of Electrical and Electronics Engineers Inc., May 2021), 7242–7252. ISBN: 9781665428125. https://arxiv.org/abs/2105.05633v3.
11. Kienholz, C., Hock, R. & Arendt, A. A new semi-automatic approach for dividing glacier complexes into individual glaciers. *Journal of Glaciology* **59,** 925–937 (Oct. 2013).
12. Lindsay, J. *WhiteboxTools* 2018. https://github.com/jblindsay/whitebox-tools.
13. Bartos, M. *Pysheds: simple and fast watershed delineation in python* 2020. https://github.com/mdbartos/pysheds.
14. Avbelj, J., Müller, R. & Bamler, R. A Metric for Polygon Comparison and Building Extraction Evaluation. *IEEE Geoscience and Remote Sensing Letters* **12,** 170–174 (2015).
15. Strozzi, T., Paul, F., Wiesmann, A., Schellenberger, T. & Kääb, A. Circum-Arctic Changes in the Flow of Glaciers and Ice Caps from Satellite SAR Data between the 1990s and 2017. *Remote Sensing* **9,** 947. ISSN: 2072-4292. https://www.mdpi.com/2072-4292/9/9/947 (9 Sept. 2017).
16. Thakur, P. K. *et al.* Gangotri glacier dynamics from multi-sensor SAR and optical data. *Advances in Space Research* **72,** 309–326. ISSN: 0273-1177. https://www.sciencedirect.com/science/article/pii/S027311772300193X (2023).
17. RGI Consortium. Randolph Glacier Inventory—A Dataset of Global Glacier Outlines, Version 7. *Boulder, Colorado USA. NSIDC: National Snow and Ice Data Center.* (2023).
18. Racoviteanu, A. E., Paul, F., Raup, B., Khalsa, S. J. S. & Armstrong, R. Challenges and recommendations in mapping of glacier parameters from space: results of the 2008 Global Land Ice Measurements from Space (GLIMS) workshop, Boulder, Colorado, USA. *Annals of Glaciology* **50,** 53–69. ISSN: 0260-3055 (53 2009).
19. Herreid, S. & Pellicciotti, F. The state of rock debris covering Earth's glaciers. *Nature Geoscience* **13,** 621–627. ISSN: 1752-0908. https://www.nature.com/articles/s41561-020-0615-0 (9 Aug. 2020).
